# Molecular landscape of etioplast inner membranes in higher plants

**DOI:** 10.1038/s41477-021-00896-z

**Published:** 2021-04-19

**Authors:** Davide Floris, Werner Kühlbrandt

**Affiliations:** Department of Structural Biology, Max Planck Institute of Biophysics, Frankfurt am Main, Germany

**Keywords:** Chloroplasts, Plant physiology

## Abstract

Etioplasts are photosynthetically inactive plastids that accumulate when light levels are too low for chloroplast maturation. The etioplast inner membrane consists of a paracrystalline tubular lattice and peripheral, disk-shaped membranes, respectively known as the prolamellar body and prothylakoids. These distinct membrane regions are connected into one continuous compartment. To date, no structures of protein complexes in or at etioplast membranes have been reported. Here, we used electron cryo-tomography to explore the molecular membrane landscape of pea and maize etioplasts. Our tomographic reconstructions show that ATP synthase monomers are enriched in the prothylakoids, and plastid ribosomes in the tubular lattice. The entire tubular lattice is covered by regular helical arrays of a membrane-associated protein, which we identified as the 37-kDa enzyme, light-dependent protochlorophyllide oxidoreductase (LPOR). LPOR is the most abundant protein in the etioplast, where it is responsible for chlorophyll biosynthesis, photoprotection and defining the membrane geometry of the prolamellar body. Based on the 9-Å-resolution volume of the subtomogram average, we propose a structural model of membrane-associated LPOR.

## Abstract (web summary)

Etioplasts are photosynthetically inactive plastids that are converted to chloroplasts under light exposure. To study thylakoid membrane biogenesis, cryo-electron tomography is used to image membranes isolated from etioplasts and reconstruct the architecture of proteins associated with the membrane.

## Main

During the past decade, electron cryo-tomography (cryo-ET) combined with subtomogram averaging has developed into a powerful technique for analysing the structure of biological assemblies in cells and organelles. In chloroplasts from higher plants^1^ and algae^2^, cryo-ET has proved to be particularly efficient for direct identification of protein complexes in mature thylakoid membranes. Electron microscopy of chloroplast precursors, known as etioplasts, has to date been limited to chemically fixed and resin-embedded material. However, neither treatment conserves the high-resolution detail required for identification of complexes or structure determination in native membranes^3–8^.

Etioplasts are well suited to the study of chloroplast development by cryo-ET. In flowering plants (angiosperms) the organelles, which are photosynthetically inactive, accumulate in etiolated seedlings grown under low light. Etioplasts are characterized by a unique inner membrane network consisting of an extensive paracrystalline mesh of tubular membranes, the prolamellar body, attached to thylakoid precursors, the prothylakoids^9^. Prothylakoids appear as disk-like planar membranes in plastic sections of leaf material or purified intact etioplasts^4–6,8,10^, but assume a characteristic vesicular shape when isolated from inner membrane fractions^3,11–15^. Following light exposure, the whole system undergoes a profound metamorphosis associated with the gradual assembly of photosynthetic membrane protein complexes and the formation of interconnected grana stacks^5,6,8^.

Numerous studies on the protein composition of membrane fractions isolated from etioplasts of *Arabidopsis*^4^, barley^16^, maize^17^, pea^5^, pine^18^ and wheat^3,19^ show that light-dependent protochlorophyllide oxidoreductase (LPOR) is probably one of the most abundant membrane-associated proteins, highly expressed in the prolamellar body^3,19,20^.

LPOR is among a handful of enzymes that are known to require light for catalysis^21^. Under physiological conditions, LPOR forms a photoactive complex with protochlorophyllide and nicotinamide adenine dinucleotide phosphate (NADPH). Light exposure triggers the reduction of protochlorophyllide to chlorophyllide, which immediately diffuses to the chlorophyll synthase where the C17 propionate is esterified with phytol as the final step in chlorophyll biosynthesis^22,23^. The photoactive complex has a photoprotective role in vitro, preventing the excited triplet state of protochlorophyllide from generating singlet oxygen^24^. Photosynthetic organisms producing chlorophyll in the absence of light rely on the phylogenetically unrelated dark-operative protochlorophyllide oxidoreductase^25^.

After extensive biochemical characterization^26–28^ Zhang et al.^21^ recently reported the first X-ray structures of LPOR from *Synechocystis* sp. and *Thermosynechococcus* *elongatus*. Even though some flexible domains are lacking, the structures suggest an overall similarity of LPOR with members of the short-chain dehydrogenase and reductase family, characterized by a central β-sheet surrounded by six main α-helices. However, little is known about the location, membrane anchoring and oligomeric state of LPOR^5,29,30^, which limits our understanding of how the enzyme functions during photomorphogenesis. LPOR has been suggested to determine the shape, preserve the architecture and control the size of the prolamellar body^31–35^, but the structural basis of these processes is unknown. Only traces of LPOR appear to be present in prothylakoids, which instead are thought to be populated by the second-most abundant complex in etioplast inner membranes, ATP synthase^3^.

In the present work, we explore the molecular landscape of etioplasts in higher plants. Building on previous studies where chemical fixation or other methodological limits prevented direct visualization and structure determination of protein complexes in native membranes, we integrate known morphological features of the etioplast inner membrane with new information about protein structure and distribution. Our tomographic volumes confirm the early accumulation of ATP synthase monomers in prothylakoids, which reflects the organization found in chloroplast stroma lamellae and grana end membranes^1^. Plastid ribosomes populate the stromal regions of the prolamellar body membrane lattice while its tubular membranes are enveloped by uniform, helical arrays of a small, membrane-associated protein. By a combination of biochemical analysis and subtomogram averaging, we identified this protein as the 37-kDa enzyme LPOR and determine its membrane-associated structure at subnanometer resolution.

## Results

### Architecture of etioplast inner membranes

Because isolated intact plastids are too thick for imaging by cryo-tomography, we used small organelles that rupture during blotting for cryo-EM specimen preparation, which enabled us to record images of minimally disturbed inner membrane networks.

**Fig. 1:**
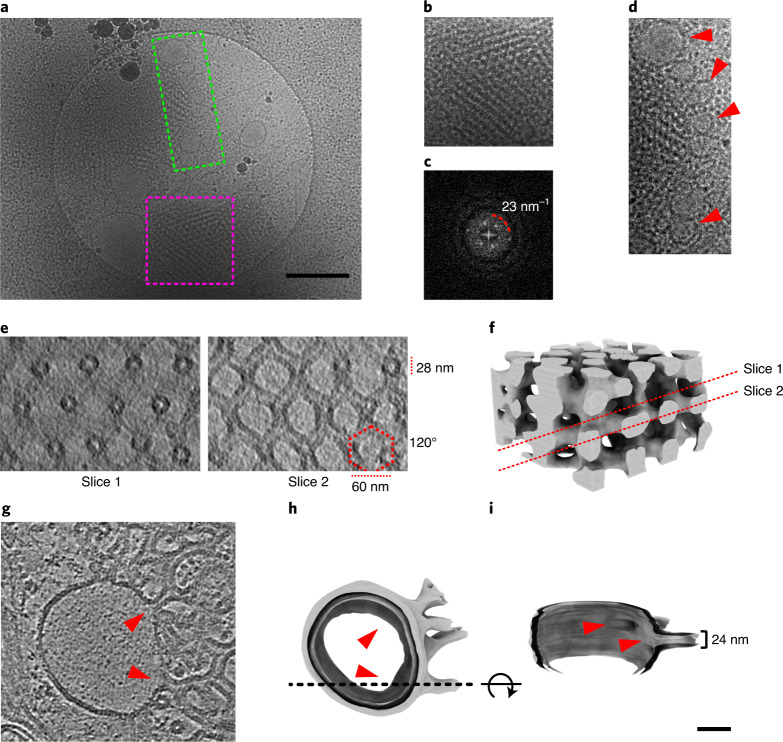
Inner membrane morphology of pea etioplasts. **a**, Low-magnification view of vitrified inner membranes. **b**–**d**, Isolated patches of the micrograph in **a** (dashed magenta square and green rectangle) highlight the morphology of prolamellar bodies (**b**), their Fourier transform (**c**) and prothylakoids (**d**, red arrowheads). **e**,**f**, Tetrahedrally branched membrane tubes are evident in consecutive slices (**e**, slices 1 and 2) through the tomographic reconstruction of a prolamellar body and its segmented surface (**f**). **g**–**i**, Tomographic section through a prothylakoid (**g**). Red arrowheads highlight circular junctions connecting the vesicular prothylakoid to the prolamellar body at positions indicated in the segmented surface (**h**) and rotated cross-section (**i**). Scale bars, 500 nm (**a**), 50 nm (**i**). Source data

At low magnification, prolamellar bodies appear as a large, regular mesh of dense regions separated by a less dense stromal space (Fig. 1a). The high degree of order is clear in the Fourier transform of selected regions (Fig. 1b,c), which extends to the second order of diffraction (~1/23 nm). The surrounding prothylakoids form large membrane vesicles (Fig. 1d) resembling those in plastic sections of isolated inner etioplast membranes^3,11–15^.

Tomographic reconstruction of minimally disturbed prolamellar bodies reveals the structure of their paracrystalline core, as seen in cross-sections at different heights of well-ordered areas (Fig. 1e,f). Membrane tubes measuring ~28 nm in width intersect at an angle of ~120° to form tetrahedral units, with a repeat distance of ~60 nm. Their assembly results in a characteristic diamond cubic lattice.

**Extended Data Fig. 1:**
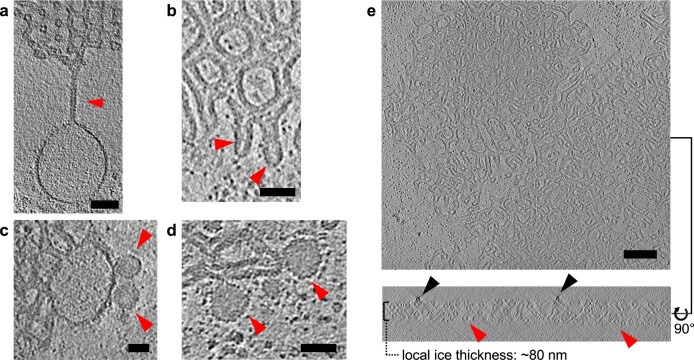
Cryo-tomography of pea etioplast inner membranes. **a**, Partly detached prothylakoid connected to the prolamellar body by a membrane tube (red arrowhead). **b**, Sealed free ends of the tubular mesh (red arrowheads). **c**, Plastoglobuli (red arrowheads) attached to prothylakoids and (**d**) tubular membranes. **e**, Disordered pea prolamellar body squeezed into a thin slab of vitrified buffer. The circular profile of multiple membrane tubes is visible in tomographic slices at a 90° angle to the grid plane (red arrowheads). Atmospheric ice contamination (black arrowheads) delineates the air-water interface. All images are slices of tomographic volumes. Scale bar size: 100 nm (a), 50 nm (b, c, d), 150 nm (e).

The regularity of the prolamellar body reduces gradually towards its periphery, where multiple tubes connect to the same prothylakoid through ~24-nm circular junctions (Fig. 1g,h). In most cases the two membrane domains are adjacent; when further apart, they are connected by single, long membrane tubes (Extended Data Fig. 1a). The free ends of tubular membranes are sealed (Extended Data Fig. 1b), creating a closed membrane compartment that encompasses the entire etioplast inner membrane. Small spherical densities that appear to be plastoglobuli are often found in the outermost leaflet of both tubules and prothylakoids (Extended Data Fig. 1c,d).

### Ribosomes and membrane-associated complexes

**Fig. 2:**
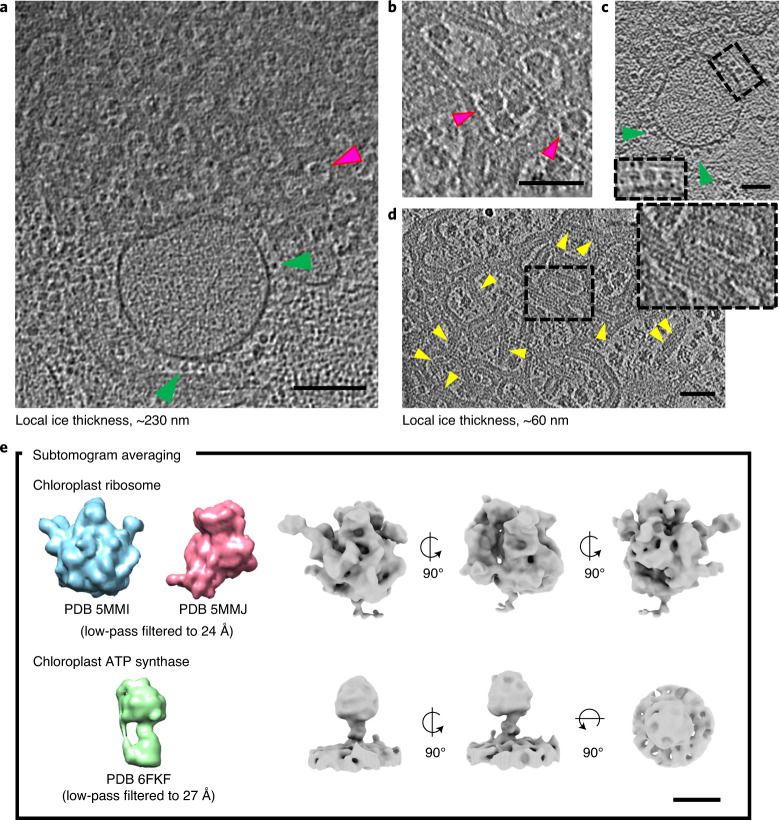
Protein complexes of prolamellar bodies and prothylakoids. **a**–**e**, Tomographic sections and subtomogram average maps. Dense globular particles (~25 nm, magenta arrowheads in **a**,**b**) are fully assembled ribosomes (**e**, top row). Lollipop-shaped particles (green arrowheads in **a,c**) are ATP synthase (**e**, bottom row). Left-handed helical arrays (**d**, yellow arrowheads and inset) decorate the tubular membranes of the prolamellar body. Scale bars, 100 nm (**a**), 50 nm (**b**–**d**), 10 nm (**e**).

Numerous large protein complexes are visible on the internal membranes of ruptured etioplasts (Fig. 2a). The largest of these are ~25 nm dense globular particles located in the stromal space of the tubular lattice (Fig. 2a,b, magenta arrowheads). A subtomogram average map at ~26-Å resolution identifies them as fully assembled ribosomes (Fig. 2e and Supplementary Fig. 1).

**Extended Data Fig. 2:**
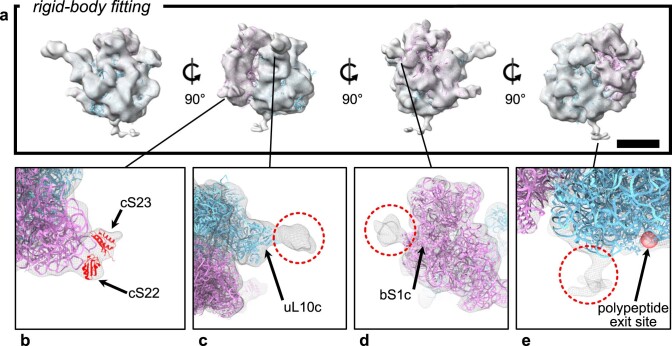
Chloroplast ribosomes in the prolamellar body. (**a**) Rigid-body fitting shows good correspondence between subtomogram average and atomic model of the 50S (PDB 5mmi, cyan) and 30S (PDB 5mmj, pink) subunit of the chloroplast ribosome. Inset (**b**) highlights the position of plastid-specific subunits cS22 and cS23 (red) and their fit in the foot region of the density map (grey mesh). Inset (**c**) and (**d**) indicate the unassigned densities (dashed red circles) connected to the uL10c and bS1c subunits. Inset (**e**) shows the location of the polypeptide exit site (red sphere), and the unassigned density (dashed red circle) next to it. Scale bar size: 10 nm.

We fitted 50S (Protein Data Bank (PDB), 5mmi) and 30S (PDB, 5mmj) subunits of chloroplast ribosomes as rigid bodies into the subtomogram averaging map (Extended Data Fig. 2a). Both subunits correlate well with the density, including the flexible foot region where chlororibosome-specific subunits cS22 and cS23 are located (Extended Data Fig. 2b). In addition, we identified three conspicuous densities that have no correspondence in the atomic models of the chlororibosome. These regions are adjacent to subunits uL10c and bS1c, and the polypeptide exit site (Extended Data Fig. 2c–e).

**Extended Data Fig. 3:**
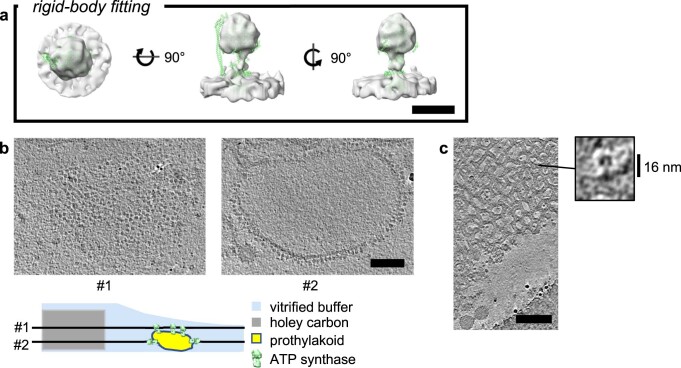
Structure and distribution of ATP synthase in prothylakoids. **a**, Rigid-body fitting of chloroplast ATP synthase (PDB 6fkf, green) to subtomogram average map shows good correspondence with the F1 head and central stalk, while the peripheral stalk is not resolved. **b**, Tomographic slices through the surface (#1) and centre (#2) of a prothylakoid vesicle indicate random distribution of ATP synthase monomers. Similar densities are occasionally found in prolamellar bodies (c). Scale bar size: 10 nm (a), 100 nm (b), 150 nm (c).

The second-largest complex we identified is a membrane protein with a diameter of ~12 nm, extending ~16 nm above the stromal side of prothylakoid membranes (Fig. 2a,c, green arrowheads and inset). Subtomogram averaging yielded a map revealing the structure of chloroplast ATP synthase (PDB, 6fki) at ~30-Å resolution (Fig. 2e, Supplementary Fig. 1 and Extended Data Fig. 3a). ATP synthase distribution on the surface of prothylakoids appeared random, without formation of dimers or higher oligomers (Extended Data Fig. 3b). Similar densities are often visible on the stromal side of tubular membranes in prolamellar bodies (Extended Data Fig. 3c), but the prominent features of the surrounding membrane tubes precluded any meaningful subtomogram averaging.

**Extended Data Fig. 4:**
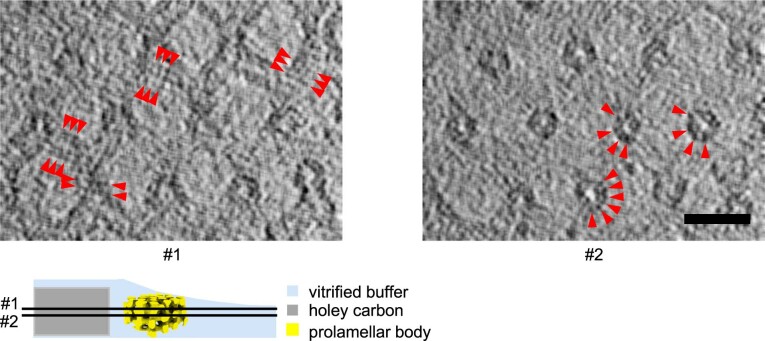
Features of tetrahedrally branched membrane tubes. Slices through the surface of the membrane tubes (#1) show seemingly regular arrays of small densities (highlighted between red arrowheads) when visualized in the unbinned tomographic reconstruction of the same ordered region shown in Fig. 1e. In cross-section (#2), the same densities are partly resolved along the circumference of the membrane tube (red arrowheads). Scale bar size: 60 nm.

Large numbers of a smaller, membrane-associated protein were identified on the membrane tubes of the paracrystalline meshwork (Fig. 2d, yellow arrowheads and inset, and Extended Data Fig. 4). This protein was most clearly visible where the mesh was squeezed into a thin (~100-nm) slab of vitrified buffer (Fig. 2d and Extended Data Fig. 1e), when it became apparent that the protein was arranged in a striking pattern of well-ordered helical arrays surrounding the tubular membranes. No such arrangement was evident in prothylakoids (Fig. 2c and Extended Data Fig. 3b).

Investigating the structure and identity of this protein in pea etioplasts was not straightforward. First, because of the small size of pea leaves, it is difficult to process sufficient plants for biochemical analysis of etioplast inner membranes in a short time; and second, the convoluted shape of appressed pea prolamellar bodies (Fig. 2d and Extended Data Fig. 1e) precluded subtomogram averaging.

Maize (*Zea mays* L.) etioplasts, on the other hand, have more desirable properties for both types of analysis. Etiolated maize seedlings produce larger leaves, and their prolamellar bodies have been reported to form straight, ~26-nm-wide tubules in response to mechanical stress. The same effect is induced by acidification below pH 6.5 and high salt content^15^.

To determine whether the surface forces acting on etioplast membranes in the thin aqueous film during cryo-EM grid preparation are sufficient for production of such elongated tubes, we repeated the cryo-EM grids with purified maize etioplasts. Possible effects of acidic pH and high salt were excluded by use of a low-salt, pH 8.0 buffer (Methods) previously shown to yield stable etioplast preparations^19^.

### Helical crystals of protochlorophyllide oxidoreductase cover prolamellar body membranes

**Fig. 3:**
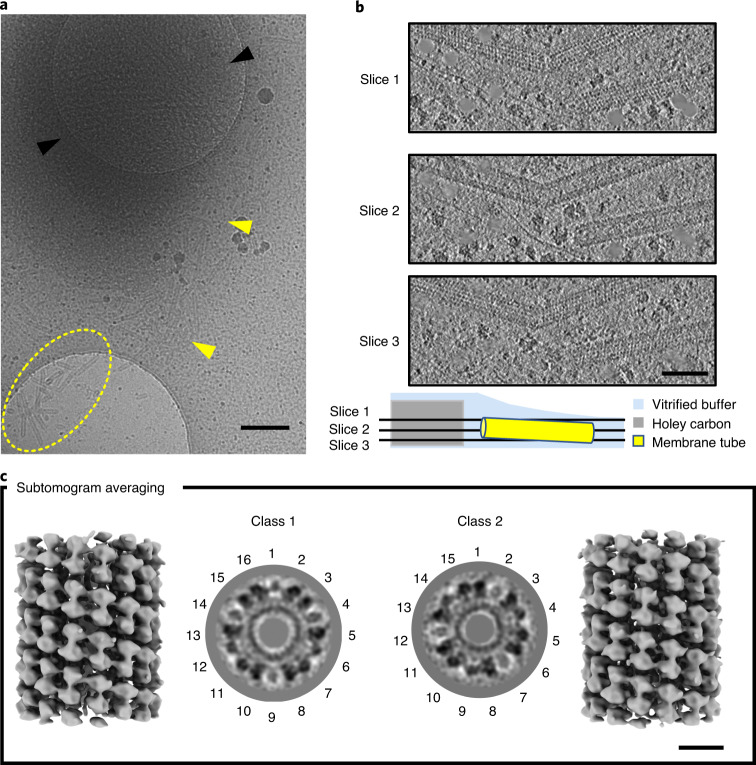
Helical arrays on membrane tubules. **a**, Overview at low magnification of compressed maize prolamellar bodies. Black arrowheads indicate the paracrystalline core of the prolamellar body; yellow arrowheads highlight a trail of straight tubular membranes connecting the core to a detached cluster (yellow dashed line). **b**, Consecutive slices through tomographic reconstruction (slices 1–3) show left-handed helical arrays identical to those in pea (Fig. 2d). **c**, Subtomogram averaging shows rows of dimers of a small, membrane-associated protein forming a left-handed helical lattice with either 16 (class 1) or 15 (class 2) units per turn, as evident in central cross-sections and lateral surface representations. Scale bars, 250 nm (**a**), 50 nm (**b**), 10 nm (**c**).

**Extended Data Fig. 5:**
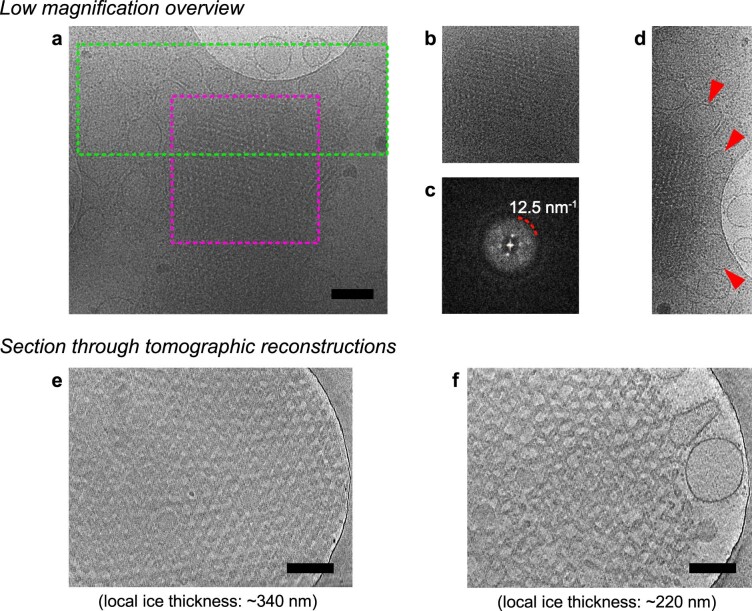
Inner membrane of maize etioplasts. (**a**) Low-magnification view of vitrified inner membranes. Isolated patches of the same micrograph (dashed magenta square and green rectangle) highlight the morphology of prolamellar bodies (**b**, and its Fourier transform, **c**) and prothylakoids (**d**, red arrowheads). Structural features closely resemble those in pea (see Fig. 1a–d). Prolamellar bodies of maize etioplasts are regular (**e**) and likewise have prothylakoids at their periphery (**f** and red arrowheads in d). Scale bar size: 250 nm (a), 150 nm (e, f).

Unperturbed inner membranes from maize etioplasts exhibit the same features as observed in pea (Extended Data Fig. 5). However, the tubules extending from compressed maize prolamellar bodies tended to be more elongated and straight, with some becoming detached and forming small clusters in the surrounding areas (Fig. 3a, yellow arrowheads and dashed oval). Their outer membrane surface was decorated by a helical pattern (Fig. 3b), much clearer than but otherwise apparently identical to that seen in pea (Fig. 2d).

**Extended Data Fig. 6:**
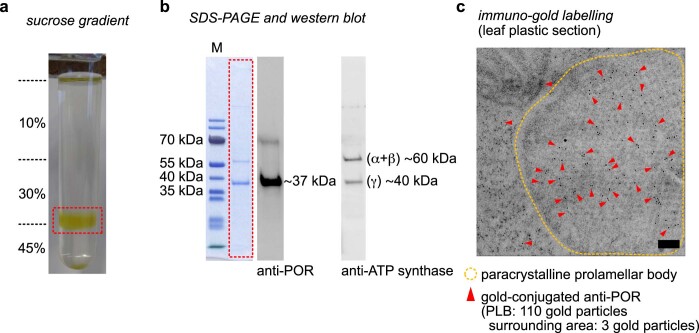
Protein composition of etioplast inner membranes. **a**, Inner membrane fraction at the 30-45% interface (red dashed rectangle) of a sucrose gradient. **b**, SDS–PAGE of the same fraction indicates a prominent band around 35 - 40 kDa, and a second band above 55 kDa, identified respectively as POR and ATP synthase subunits by Western blot analysis. M, molecular markers. **c**, Gold-conjugated anti-POR antibodies (red arrowheads) decorate the paracrystalline prolamellar body (orange dashed line). Unspecific labelling is negligible. Scale bar size: 250 nm.

On a sucrose density gradient, homogenized maize etioplasts separated into a lighter band of inner membranes and a heavier pellet of intact organelles and starch (Extended Data Fig. 6a). SDS–polyacrylamide gel electrophoresis (SDS–PAGE) of the lighter fraction indicated the presence of one predominant species with an apparent molecular weight of ~37 kDa (Extended Data Fig. 6b); immunoblot analysis identified this band as LPOR. In the same way, the second-most abundant component detected in SDS gels was attributed to the α- and β-subunits of ATP synthase (Extended Data Fig. 6b), in agreement with previous reports^3,19^. Immuno-gold labelling of leaf plastic sections with anti-LPOR antibodies resulted in the accumulation of gold beads on the paracrystalline core of the prolamellar body, which were largely absent in the surrounding areas (Extended Data Fig. 6c), indicating that the protein lattice decorating the membrane tubes is indeed LPOR.

**Extended Data Fig. 7:**
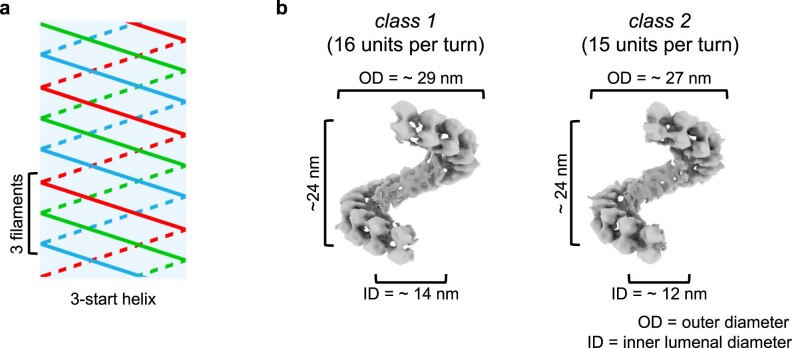
Details of helical arrays. **a**, Graphical representation of a 3-start helix. **b**, Size comparison between single helix turns of class 1 and class 2 subtomogram averages (Fig. 3c).

Subtomogram averaging shows that LPOR forms rows of dimers that wrap around the tubes in a left-handed, three-start helical lattice (Fig. 3c), with three parallel helical filaments of dimers and a ~24-nm pitch (Extended Data Fig. 7). Multi-reference alignment classification detected small differences in helix diameter. A total of 1,678 subvolumes fell into two main classes, featuring particles with either 16 (class 1) or 15 (class 2) units per turn (Fig. 3c). The outer and inner helix diameter of class 1 was, respectively, ~29 and ~14 nm, whereas in class 2 both dimensions were narrower by ~2 nm (Extended Data Fig. 7b). The two reconstructions were refined independently to 18.2- and 18.1-Å resolution, respectively (Supplementary Fig. 2), and used as references to improve local alignment in subsequent rounds of refinement.

### Subnanometer-resolution structure of membrane-associated LPOR

**Fig. 4:**
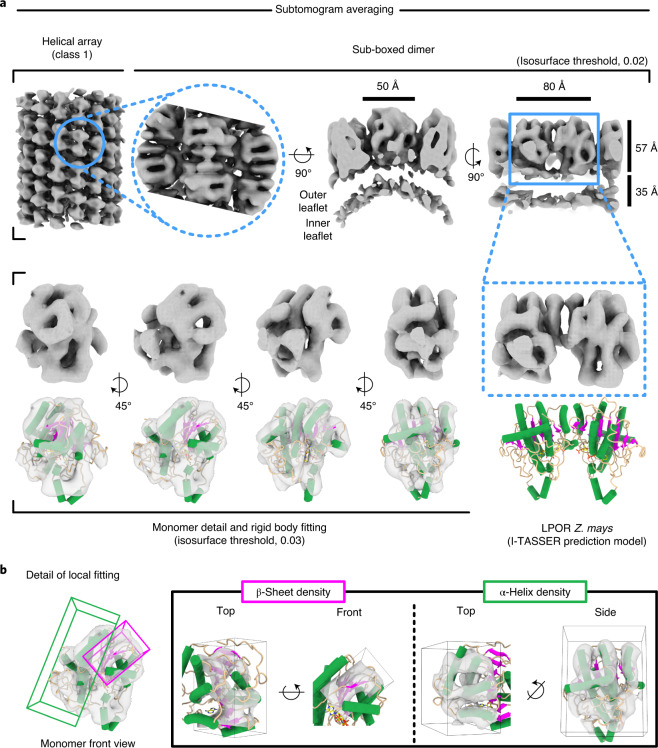
Structure of membrane-bound LPOR. **a**, Subtomogram average of class 1 sub-boxes. Top view and slice through side view indicate the size and orientation of the LPOR dimer on the membrane at an isosurface threshold of 0.02. At a higher contour level (isosurface, 0.03), four different map views show the structural features of a single LPOR monomer and their close agreement with the model of maize LPOR generated by I-TASSER. **b**, Close-up view of the elements of secondary structure. The β-sheet fits into a wide, flattened density; the α-helices fit into elongated, rod-shaped densities.

**Fig. 5:**
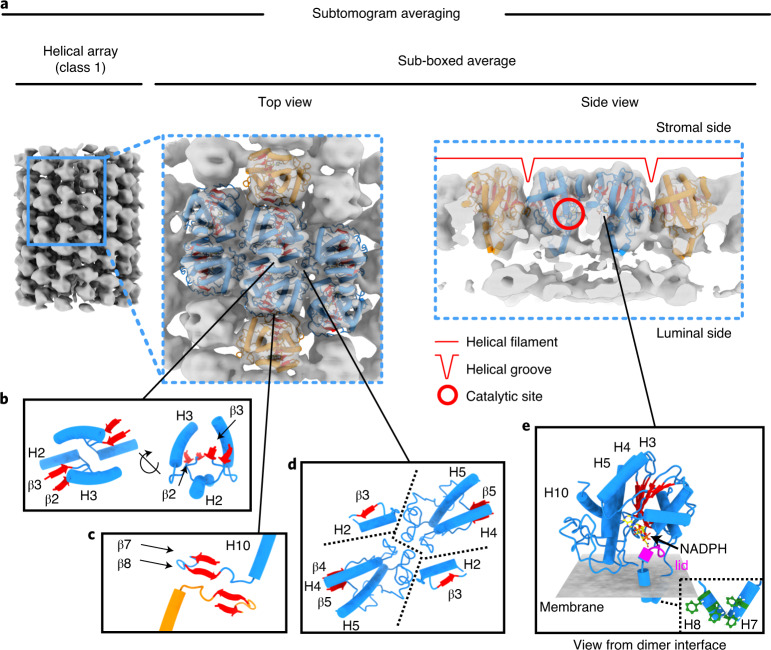
Membrane interaction and molecular contacts of LPOR monomers. **a**, Rigid-body fitting of maize LPOR model to the subtomogram average map. Blue models refer to the central helical filament, orange models to neighbouring filaments. **b**–**d**, interaction sites at the dimer interface (**b**), the interface between dimer rows (**c**) and between dimers (**d**). Dashed lines in **d** separate secondary structure elements from different monomers. **e**, Detail of protein–membrane interaction, with membrane surface in grey, lid domain (residues 246–252) in magenta and NADPH in yellow. The inset indicates the position of four Phe residues (green) likely to promote membrane interaction. H, helix.

**Extended Data Fig. 8:**
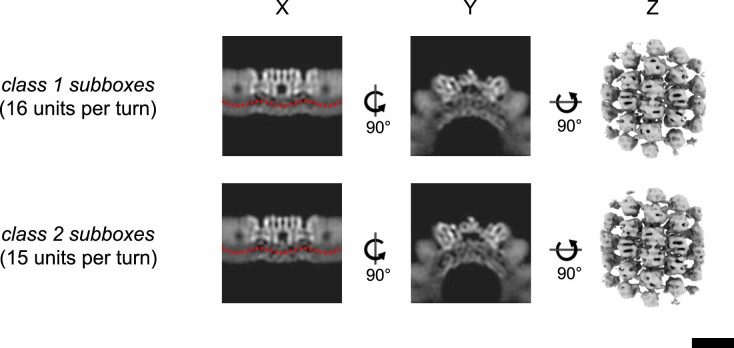
Comparison of class 1 and class 2 subboxes. Central section of side views (X and Y), and isosurface of top view (Z) of subtomogram averages from class 1 and 2 subboxes. Dashed red lines trace the outer membrane surface, highlighting negative membrane curvature below each dimer. Scale bar size: 80 Å.

**Extended Data Fig. 9:**
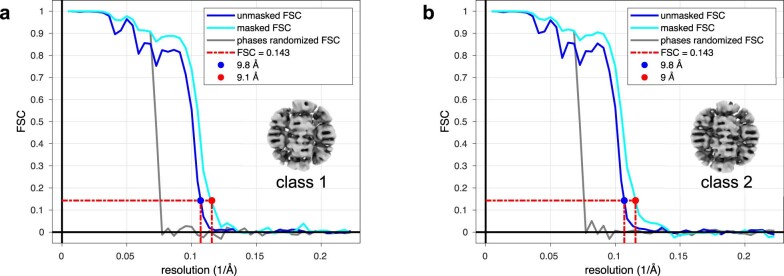
Resolution of subtomogram averages of membrane tube subboxes. Fourier shell correlation of unfiltered half-maps from class 1 (**a**) and class 2 (**b**). Map resolutions are 9.1 Å (class 1), and 9 Å (class 2).

Particles in classes 1 and 2 were sub-boxed along the helical path, then processed separately. Because the resulting maps were largely identical, it is sufficient to describe the model from class 1 only (Figs. 4 and 5 and Extended Data Fig. 8). LPOR forms symmetrical dimers with overall dimensions of ~80 × ~50 Å, extending to a height of ~57 Å above the membrane surface (Fig. 4a). Slices along the helix axis indicate a negative (concave) curvature of the membrane where it interacts with the LPOR dimers (dashed red lines in Extended Data Fig. 8). The quality of the reconstructions, both at a resolution of 9 Å (Extended Data Fig. 9), allows identification of partly resolved α-helices and β-sheets (Fig. 4).

**Extended Data Fig. 10:**
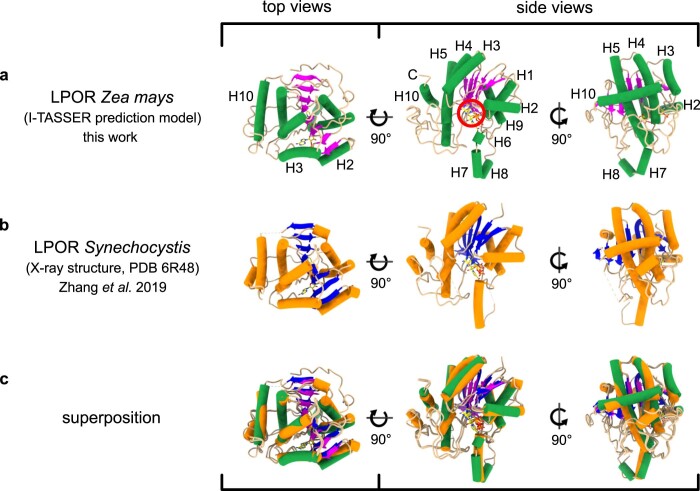
Atomic models of LPOR. Maize model generated by I-TASSER fitted to the subtomogram average volume (**a**) and X-ray structure from *Synechocystis* (**b**) agree closely (**c**).

We used the programme I-TASSER^36^ to construct a three-dimensional (3D) model of LPOR based on the *Zea mays* primary sequence (Methods). Among multiple structural templates in the PDB, the programme selected the X-ray structure of *Synechocystis* LPOR (PDB: 6r48 (ref. ^21^)) as the best fit and generated a template-based structural model (Fig. 4a and Extended Data Fig. 10).

The model of maize LPOR features a central, eight-stranded β-sheet surrounded by ten α-helices (Extended Data Fig. 10a). A NADPH molecule was placed within the catalytic pocket (red circle in Fig. 5a and Extended Data Fig. 10a), because the X-ray structure of cyanobacterial LPOR shows NADPH bound in this position. The protochlorophyllide substrate was not modelled. Rigid-body fitting (Figs. 4 and 5a) indicates a high correlation factor of 0.9 between the in silico model and our subtomogram average density map. The β-sheet and most helices of the protein align vertically on top of the membrane (Figs. 4 and 5a and Extended Data Fig. 8). No transmembrane segment is evident. Anchoring to the outer leaflet of the bilayer appears to be mediated by helices 7 and 8.

At the dimer interface, residues in the links from β-strand 2 to helix 2 and β-strand 3 to helix 3 closely approach the corresponding residues of the opposite monomer (Fig. 5b). The flexible regions linking β-strand 4 to helix 4 and β-strand 5 to helix 5 stretch sideways towards the long side of the next dimer, contacting as indicated in Fig. 5d. Minor contacts are also evident across dimers on neighbouring helical filaments, suggesting an interaction between segments joining β-strand 8 to helix 10 on either side of the helical groove (Fig. 5c).

## Discussion

We used cryo-ET to study the supramolecular organization of protein complexes in frozen-hydrated inner membranes of etioplasts from pea and maize. The etioplast membrane systems were readily observable on EM grids when the organelles were disrupted by blotting immediately before plunge-freezing. This approach, introduced by Daum et al.^1^, preserves lateral membrane heterogeneity and native protein composition. Previous studies with plastic sections of chemically fixed organelles and leaves^6–8^, although unable to reveal molecular detail, are broadly consistent with our cryo-tomograms of unfixed, flash-frozen organelles. By contrast, our cryo-tomograms and subtomogram averages show the 3D structures of etioplast membrane protein complexes directly in the native membranes.

Prothylakoids are characterized by low curvature, absence of appressed regions and a high percentage of the bilayer-forming lipid digalactosyldiacylglycerol. In this respect they resemble chloroplast stroma lamellae and grana end membranes. Vesicular prothylakoids are frequently found in preparations of isolated etioplast inner membranes^3,11,12,14,15^. In our specimens (Fig. 1a and Extended Data Fig. 5a), this morphology is probably due to slight osmotic swelling before or during plunge-freezing, which is an unavoidable consequence of the reduced osmolarity of the buffers used for vitrification. A low concentration of osmolytes, such as sugars and salts, is mandatory to warrant sufficient contrast for cryo-EM analysis.

ATP synthase is distributed uniformly in prothylakoid membranes (Fig. 2a,c and Extended Data Fig. 3b). Its monomeric state, evident in our tomographic reconstructions (Extended Data Fig. 3b), is in line with cryo-tomographic studies of mature thylakoid membranes^1^ and native gel electrophoresis^16^. Occasional lollipop-shaped membrane protein complexes found in the tubular lattice of the prolamellar body (Extended Data Fig. 3c) suggest that this membrane region also contains some ATP synthase, consistent with an earlier biochemical analysis of isolated etioplast inner membrane fractions^3^.

From a functional perspective, the operational framework of etioplast ATP synthase is affected by the absence of a fully assembled photosynthetic electron transport chain. In a recent study, Kambakam et al.^37^ suggested that the chemiosmotic requirements for ATP synthesis in etioplasts may be satisfied by the proton-pumping activity of NAD(P)H dehydrogenase (NDH) and cytochrome *b*_*6*_*/f* (Cyt *b*_*6*_*/f*). This is proposed as part of a larger pathway similar to mitochondrial oxidative phosphorylation, also found in other photosynthetically inactive plastids (reviewed in Renato et al.^38^). Apart from ATP synthase, none of the other protein complexes involved are resolved in our tomograms, most probably because of their lower molecular mass or the smaller size of their solvent-exposed domains.

Large globular densities are occasionally found in the stromal region within the tubular lattice of prolamellar bodies (Fig. 2a,b). Earlier work referred to these as ‘ribosome-like particles’, because they were degraded by ribonuclease^11^. Our subtomogram averaging map (Fig. 2e and Extended Data Fig. 2) provides direct evidence that they are mature chloroplast ribosomes. At the current resolution (~30 Å), the three conspicuous density regions that are absent from the atomic model of Bieri et al.^39^ cannot be identified unambiguously. Previous studies, however, report structures with similar features that may shed light on their identity and function.

The most prominent of these three regions is an arc-shaped density protruding from the 50S uL10c subunit (Extended Data Fig. 2c). L7/L12 oligomers bound to bacterial ribosomes share the same feature^40^. These proteins form a flexible stalk that is thought to facilitate the recruitment of translation factors and guanosine triphosphate hydrolysis. The second region is attached to bS1c in the 30S subunit (Extended Data Fig. 2d), a flexible protein that plays a central role in messenger RNA binding and translation initiation and is only partially resolved in the atomic model^39^. One of its oligonucleotide-binding folds protrudes towards the mRNA exit channel. The other two oligonucleotide-binding folds are not identified, and may account for some of the unassigned density in our subtomogram average (Extended Data Fig. 2d, dashed red line). The third region is a small density near the polypeptide exit site (Extended Data Fig. 2e), similar to the ribosomal RNA loop that stabilizes membrane interaction during cotranslational translocation in bacterial ribosomes^41^. Further investigation should resolve the molecular details of these regions, and provide insight into the activation state and membrane association of etioplast ribosomes.

LPOR assembles into highly ordered helical arrays that decorate the surface of prolamellar bodies (Figs. 2d and 3b and Extended Data Fig. 4). This observation explains the direct correlation between the size of the paracrystalline membrane network and LPOR expression levels^32–34^. Additionally, it explains the recent findings of Yamamoto et al.^42^, who showed that overexpression of LPOR in etiolated cyanobacteria produces large membrane structures that look like prolamellar bodies. Isolated membrane tubes (Fig. 3 and Extended Data Fig. 7b) present the same diameter as those in the meshwork of the prolamellar body (Fig. 1e), as previously observed by Selstam et al.^15^. They appear, however, to be straighter and longer, possibly as a consequence of etioplast rupture during blotting. Further investigation under experimental conditions that do not generate mechanical stress (for example, by examining focused ion beam-milled lamellae of vitrified plant cells) should clarify these aspects.

By comparison, the diameter of prothylakoid junctions was narrower by ~4 nm (Fig. 1g,h). This may signify a loss of order in the LPOR helical arrays, or a transition in protein composition between the two membrane domains. The regular decoration with LPOR protein is not evident in prothylakoids, but the presence of a smaller pool of less ordered complexes cannot be ruled out as these would be harder to identify.

Experiments based on cross-linking, fluorescence and circular dichroism spectroscopy of both native membranes and reconstituted photoactive complexes have previously been performed, suggesting that LPOR might form large aggregates^43–45^, but nothing was known about their arrangement and order. Our cryo-ET results provide direct evidence of array formation in vivo and greatly expand the model proposed by Reinbothe and co-authors^29,46^, where heterologous expression and in vitro reconstitution with lipids yielded LPOR hexamers dubbed light-harvesting POR:protochlorophyllide supercomplexes.

According to our model, the residues that mediate monomer interaction at the dimer interface are probably located in helices 2–3, β-strands 2–3 or the flexible loops connecting them (Fig. 5b). Deletion of the ‘extra loop’, a small region distinguishing LPOR from the structurally related alcohol dehydrogenases, was shown to impair LPOR oligomer formation^47^. This loop, corresponding to Gly166-Asp201 in maize LPOR, is located upstream of helix 5 and appears to mediate a lateral contact between adjacent dimers (Fig. 5d).

The effect of individual LPOR dimers on membrane morphology is evident from the local negative curvature observed in our subtomogram averages (Fig. 4a and Extended Data Fig. 8). Based on the configuration determined by rigid-body fitting of the predicted model from maize (Figs. 4 and 5a), we propose that helices 7 and 8 might stabilize interaction with the bilayer through four Phe residues (259, 263, 266 and 269; Fig. 5e). In cyanobacterial LPOR, the corresponding residues were suggested to be part of a large hydrophobic patch that plays a role in catalytic activity, protochlorophyllide binding and the formation of higher-order assemblies^48^.

Other than by protein interaction, negative membrane curvature might result from the physicochemical properties of monogalactosyldiacylglycerol (MGDG), the most abundant lipid in the prolamellar body^12^. MGDG has an intrinsic tendency to form inverse hexagonal lipid phases, which would create negative curvature and elastic stress, and exert lateral pressure on proteins embedded in the lipid bilayer. Recent evidence suggests that MGDG regulates LPOR allosterically, inducing conformational changes with a strong effect on catalytic efficiency^45^. Conversely, the absence of MGDG is detrimental to the formation and oligomerization of the photoactive LPOR complex^10^. Photoprotective carotenoids such as lutein might play an equally important role, since these are known to influence the morphology of the prolamellar body and its interaction with LPOR^49^.

**Fig. 6:**
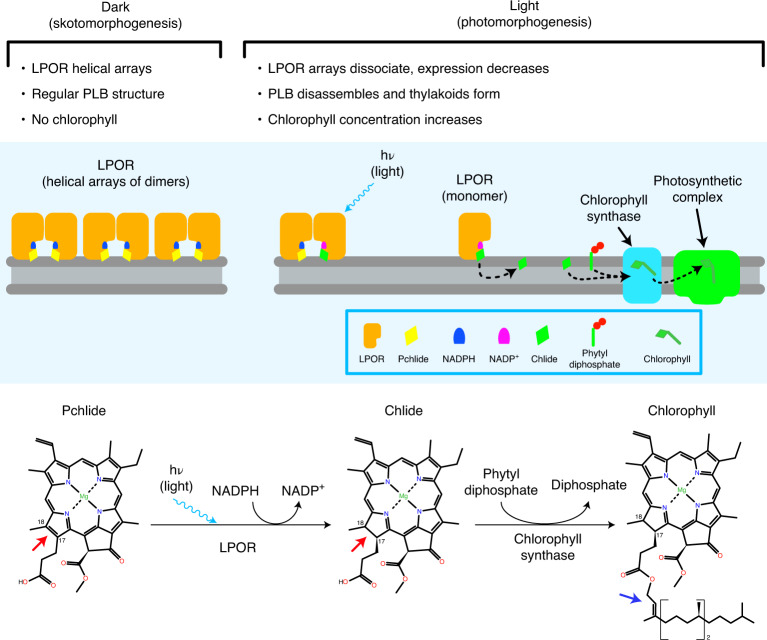
Proposed mechanism for chlorophyll biosynthesis in plastid membranes. Under dark conditions, helical LPOR arrays preserve the compact, regular architecture of the prolamellar body (PLB) while storing a ready-to-use pool of protochlorophyllide (Pchlide). Light exposure triggers LPOR activation and the PLB disassembles. LPOR converts Pchlide to chlorophyllide (Chlide), which diffuses in the membrane to the chlorophyll synthase. Chlide is esterified with a phytol chain to produce chlorophyll, which is immediately bound by a photosynthetic complex. Red arrows in the lower panel highlight the C17–C18 bond reduced by LPOR. The blue arrow indicates the position of the phytol chain esterified by the chlorophyll synthase.

In LPOR from cyanobacteria^21^ a loop between residues 223 and 229 acts as a lid for the catalytic site. In maize, this region corresponds to residues 246–252, which are part of helix 6 and the loop connecting it to β-strand 6 (magenta in Fig. 5e). Our analysis suggests that, in the physiological context of the prolamellar body, LPOR assumes an optimal orientation to facilitate the swift exchange of protochlorophyllide and chlorophyllide between its catalytic site and the outer membrane leaflet (Figs. 5e and 6). This would be in agreement with the amphipathic character of protochlorophyllide, which would make it compatible with the lipid head groups of the outer leaflet and promote its uptake from LPOR^50,51^.

Sequestration of protochlorophyllide by LPOR has been suggested to prevent generation of photo-oxidized toxic forms of the pigment^22^. The tight packing and uniform distribution of LPOR on the etioplast inner membranes leaves room for speculation about a more elaborate photoprotective system along the lines of a resonance–energy transfer mechanism between neighbouring LPOR complexes^29,46^. This might resemble the carotenoid- and chlorophyll-based photoprotective mechanisms that are well characterized in photosystems and light-harvesting complexes of mature thylakoids^52^.

## Methods

### Etioplast isolation

Pea and maize seedlings were grown in a darkened growth chamber at a constant temperature of 20 °C for 7 and 13 days, respectively. Etioplasts were isolated according to Blomqvist et al.^19^ with minor modifications. Briefly, leaves were ground with a Waring blender in ice-cold buffer A (pea: 50 mM Tricine/NaOH pH 7.5, 300 mM sorbitol, 10 mM CaCl_2_, 10 mM MgCl_2_; maize: 20 mM HEPES/NaOH pH 8.0, 300 mM sorbitol, 10 mM NaHCO_3_, 5 mM MgCl_2_), then filtered through four layers of Miracloth (Merck). The organelles were pelleted (4,500*g*, 15 min, 4 °C), resuspended and further purified by centrifugation (4,500*g*, 20 min, 4 °C) on a discontinuous 40–80% Percoll gradient (Merck) in buffer A. Intact etioplasts were isolated from the 40–80% interface and washed twice in buffer A to eliminate residual Percoll. All steps were carried out in the dark or under safe green light.

### Biochemical analysis of etioplast inner membranes

Inner membranes were extracted from intact maize etioplasts by homogenization with a glass Potter homogenizer, then isolated from the 30–45% interface of a discontinuous 10/30/45% sucrose gradient in buffer A by centrifugation (25,400*g*, 2 h, 4 °C). Protein composition was assessed by SDS–PAGE as previously described by Haniewicz et al.^53^, without modification. For immunoblot analysis, proteins were transferred on a polyvinylidene difluoride membrane with a Trans-Blot Turbo system and RTA Transfer Kit (Bio-Rad) according to the manufacturer’s instructions. Anti-POR (no. AS05 067, dilution 1:2,000) and anti-ATP synthase (no. AS08 370, dilution 1:10,000) were used as primary antibodies (Agrisera), and goat anti-rabbit (no. 4055-05, dilution 1:1,000) as the secondary antibody (SouthernBiotech). The horseradish peroxidase signal was generated with the ECL Start Western Blotting Reagent kit (GE Healthcare) and detected with a ChemiDoc Touch Imaging System (Bio-Rad). Full scans of SDS–PAGE gel and immunoblots are available at Source Data Fig. 1.

For immuno-gold labelling, fresh sections of etiolated leaves were placed on a glass slide and gently sliced with a scalpel, then treated as described in Wilkes et al.^54^ with minor modifications. Briefly, after fixation with 4% paraformaldehyde in PBS, the sample was dehydrated in an ethanol-gradient series and infiltrated with LR white medium-grade acrylic resin (London Resin). After 48 h of polymerization at 65 °C, the pellets were cut into 60–80-nm slices with an Ultracut S Microtome (Leica) and deposited on gold grids (Plano) coated with a layer of amorphous carbon and Formvar. Labelling was carried out with the same anti-POR primary antibody (dilution 1:200) as used for imunoblots, but gold-conjugated AffiniPure goat anti-rabbit (Jackson ImmunoResearch) was used as secondary antibody (no. 111-205-144, dilution 1:20). Samples were imaged in a Tecnai G2 Spirit BioTwin (FEI).

### Cryo-EM grid preparation

Immediately before vitrification, fresh intact etioplasts were pelleted for 3 min with a benchtop centrifuge then resuspended in half the initial volume of cryo buffer (similar to buffer A, but with sorbitol replaced by 150 mM trehalose). At this point the absence of chlorophyll, used as marker of accidental light exposure, was monitored by acetone extraction as described by Haniewicz et al.^55^. Organelles were mixed 3:1 with 6- or 10-nm gold fiducials (AURION Immuno Gold Reagents & Accessories), then 3 µl of suspension was immediately applied to glow-discharged Quantifoil R0.6/1 or R2/2 300 mesh copper grids (Quantifoil Micro Tools) and plunge-frozen with a Vitrobot Mark IV (FEI) after blotting for 6–8 s at 100% relative humidity.

### Cryo-ET

Data were collected with a Titan Krios electron microscope (FEI) operated at 300 kV in energy-filtered transmission electron microscopy mode, as previously described in D’Imprima et al.^56^. The magnification was chosen to achieve a calibrated pixel size of 2.2 Å. Tilt series were collected from +60° to –60° using a dose-symmetric acquisition scheme^57^ implemented in SerialEM^58^. The acquisition parameters are summarized in Supplementary Tab. 1. Dose-fractionated images were recorded in counting mode on a K2 summit or K3 direct electron detector (Gatan), then motion corrected and dose weighted with MotionCor2 (ref. ^59^). Contrast transfer function correction and reconstruction with weighted back-projection were done in IMOD^60^. All tomographic images were slices ten pixels in thickness. Contrast was enhanced with either a nonlinear anisotropic diffusion filter^61^ or a 3D median filter in MATLAB (MathWorks). Volumes were segmented manually with a combination of the IMOD drawing tool plug-in and the EMAN convolutional neural network method^62^.

### Subtomogram averaging and homology modelling

Subtomogram averaging was performed with Dynamo^63^ according to the gold standard procedure^64^. Chlororibosomes and ATP synthase subvolumes were extracted from seven tomograms. For the chlororibosome dataset, 315 particles within the tubular lattice of prolamellar bodies were picked manually while 377 ATP synthase complexes were selected from the neighbouring prothylakoids. Isolated ribosomes and ATP synthase complexes on membranes detached from prolamellar bodies were excluded from the analysis. In both datasets, 20 random particles were used as initial reference and the final maps were bandpass filtered to 26 Å for ribosomes and 30 Å for ATP synthase.

For LPOR averaging, membrane tubes from 17 tomograms were sampled at regular intervals along their axis with the addModPts function in PEET^65^. Multi-reference alignment, performed as in D’Imprima et al.^56^, split them in two main classes of 950 and 728 particles featuring either 16 (class 1) or 15 (class 2) helical repetitions per turn, respectively. Independent processing yielded reconstructions at 18.2- and 18.1-Å resolution. Both maps were bandpass filtered to the respective nominal resolution and used to find the coordinates of individual LPOR dimers along the helical path. A total of 20,082 and 17,080 sub-boxes were extracted from particles in classes 1 and 2, respectively, then aligned in Dynamo. For both datasets, a further subtilt refinement step was performed. Using the dyn2rel package^66^, 3D sub-boxes contributing to each map were automatically traced back to their two-dimensional projections, cropped from each image of the tilt series and finally processed with Relion-3 (ref. ^67^). Both maps attained a final resolution of 9 Å.

Structural features were compared to a 3D model generated with I-TASSER^36^ from the primary sequence of *Z. mays* LPOR (GenBank: PWZ38724.1) based on the X-ray structure of *Synechocystis* LPOR (PDB: 6r48). The 50-amino acid transit peptide at the N terminus was excluded. All final maps were displayed with ChimeraX^68^.

### Statistics and reproducibility

Etioplasts from pea and corn were analysed from 28 and 11 independent preparations, respectively. The micrographs, tomographic reconstructions and experimental results in all figures are representative of at least three independent replicates. The tomograms used for subtomogram averaging analysis were collected on grids prepared from three (pea) and five (maize) independent purifications. All attempts to reproduce the results were successful.

### Reporting Summary

Further information on research design is available in the Nature Research Reporting Summary linked to this article.

## Supplementary information

Supplementary Information Supplementary Figs. 1 and 2 and Table 1.

Reporting Summary

## Data Availability

The cryo-EM maps were deposited in the Electron Microscopy Data Bank with accession codes EMD-11959 (ribosome), EMD-11958 (ATP synthase), EMD-11961 (LPOR in membrane tubes from class 1), EMD-11960 (LPOR in membrane tubes from class 2), EMD-11963 (LPOR class 1 sub-boxes) and EMD-11962 (LPOR class 2 sub-boxes). Source data are provided with this paper.

## Source data

Source Data Fig. 1 Unprocessed immunoblots and gels.
