# Supplementary material for: Molecular landscape of etioplast inner membranes in higher plants
Source: Nat Plants. 2021 Apr 19;7(4):514–23. doi: 10.1038/s41477-021-00896-z (PMC8055535; doi:10.1038/s41477-021-00896-z)
Supplement: Supplementary file 1 — Supplementary Figs. 1 and 2 and Table 1. [file 41477_2021_896_MOESM1_ESM.pdf]

nature portfolio

https://doi.org/10.1038/s41477-021-00896-z

Supplementary information

Molecular landscape of etioplast inner membranes in higher plants

In the format provided by the authors and unedited

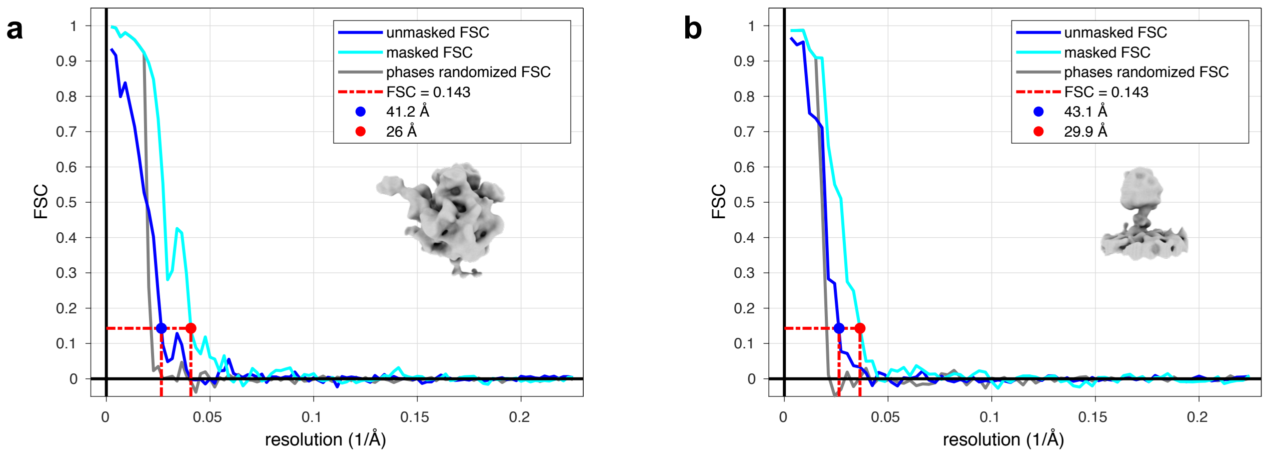

Supplementary Figure 1 | Map resolution of subtomogram averages.

Fourier shell correlation of unfiltered half maps of ribosomes (a) and ATP synthase (b). Map resolutions are 26 Å (ribosome) and 29.9 Å (ATP synthase).

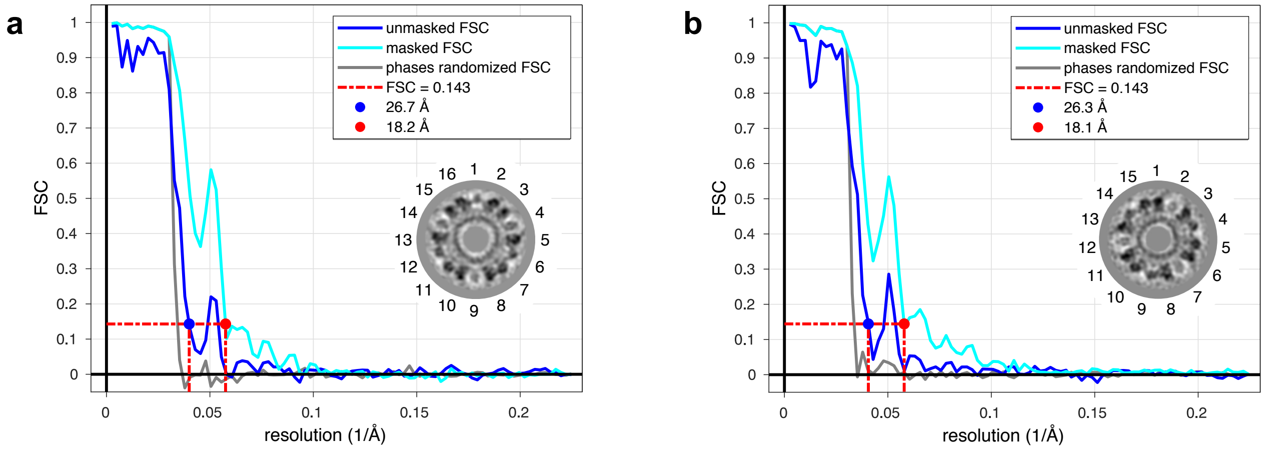

Supplementary Figure 2 | Resolution of subtomogram averages of membrane tube segments.

Fourier shell correlation of unfiltered half maps from class 1 (a) and class 2 (b). Map resolutions are 18.2 Å (class 1), and 18.1 Å (class 2).

	ATP synthase (EMD-11958)	Ribosome (EMD-11959)	LPOR helical arrays (class 1) (EMD-11961)	LPOR helical arrays (class 2) (EMD-11960)	LPOR subboxed helical arrays (class 1) (EMD-11963)	LPOR subboxed helical arrays (class 2) (EMD-11962)
Voltage (kV)	300	300	300	300	300	300
Camera/detector	K2	K2	K2	K2	K2	K2
Magnification	64,000	64,000	64,000	64,000	64,000	64,000
Pixel size (Å)	2.2	2.2	2.2	2.2	2.2	2.2
Target defocus (µm)	5	5	2.5	2.5	2.5	2.5
Tilt range (step)	±60 (2°)	±60 (2°)	±60 (3°)	±60 (3°)	±60 (3°)	±60 (3°)
Exposure time (s/image)	1.25	1.25	1.5	1.5	1.5	1.5
No. movie frames	5	5	0.5	0.5	0.5	0.5
Total exposure ($e^-/Å^2$)	150	150	90	90	90	90
No. tomograms	4	4	17	17	17	17
No. particles	377	315	950	728	20082	17080
Symmetry	C1	C1	C1	C1	C1	C1
Resolution (Å)	29.9	26	18.2	18.1	9.1	9
Validation method	FSC (gold standard)	FSC (gold standard)	FSC (gold standard)	FSC (gold standard)	FSC (gold standard)	FSC (gold standard)

Supplementary Table 1 | Tomographic data acquisition and processing.
