# Supplementary material for: Molecular landscape of etioplast inner membranes in higher plants
Source: Nat Plants. 2021 Apr 19;7(4):514–23. doi: 10.1038/s41477-021-00896-z (PMC8055535; doi:10.1038/s41477-021-00896-z)
Supplement: Supplementary file 2 — Reporting Summary [file 41477_2021_896_MOESM2_ESM.pdf]

nature research

Corresponding author(s): Werner Kühlbrandt, Davide Floris

Last updated by author(s): Feb 11, 2021

Reporting Summary

Nature Research wishes to improve the reproducibility of the work that we publish. This form provides structure for consistency and transparency in reporting. For further information on Nature Research policies, see our Editorial Policies and the Editorial Policy Checklist.

Statistics

For all statistical analyses, confirm that the following items are present in the figure legend, table legend, main text, or Methods section.

n/a	Confirmed	
☐	☒	The exact sample size (*n*) for each experimental group/condition, given as a discrete number and unit of measurement
☐	☒	A statement on whether measurements were taken from distinct samples or whether the same sample was measured repeatedly
☒	☐	The statistical test(s) used AND whether they are one- or two-sided<br>*Only common tests should be described solely by name; describe more complex techniques in the Methods section.*
☒	☐	A description of all covariates tested
☒	☐	A description of any assumptions or corrections, such as tests of normality and adjustment for multiple comparisons
☒	☐	A full description of the statistical parameters including central tendency (e.g. means) or other basic estimates (e.g. regression coefficient) AND variation (e.g. standard deviation) or associated estimates of uncertainty (e.g. confidence intervals)
☒	☐	For null hypothesis testing, the test statistic (e.g. *F*, *t*, *r*) with confidence intervals, effect sizes, degrees of freedom and *P* value noted<br>*Give P values as exact values whenever suitable.*
☒	☐	For Bayesian analysis, information on the choice of priors and Markov chain Monte Carlo settings
☒	☐	For hierarchical and complex designs, identification of the appropriate level for tests and full reporting of outcomes
☒	☐	Estimates of effect sizes (e.g. Cohen's *d*, Pearson's *r*), indicating how they were calculated

Our web collection on statistics for biologists contains articles on many of the points above.

Software and code

Policy information about availability of computer code

Data collection	SerialEM v3.6 (described in: Mastronarde, D. N. 2005. J Struct Biol, 152: 36-51).
Data analysis	Dynamo v1.1.253 (described in: Castano-Diez, D. 2017. Acta Crystallogr D Struct Biol, 73: 478-87).<br>dyn2rel package (described in: Sanchez, R. M., et al. 2020. Nat Commun, 11: 3709).<br>MATLAB vR2019a (The MathWorks Inc.)<br>MotionCor2 v1.2.6 (described in: Zheng, S. Q., et al. 2017. Nature Methods, 14: 331-32).<br>IMOD v4.8.56 (described in: Kremer, J. R., et al. 1996. Journal of Structural Biology, 116: 71-76).<br>EMAN v2.31 (described in: Chen, M., et al. 2019. Nature Methods, 16: 1161-+).<br>PEET v1.10.0 (described in: Nicastro, D., et al. 2006. Science, 313: 944-8).<br>RELION v3.1.0 (described in: Zivanov, J., et al. 2018. Elife, 7).<br>I-TASSER (described in: Yang, J. Y., and Y. Zhang. 2015. Nucleic Acids Research, 43: W174-W81).<br>ChimeraX v0.93 (described in: Goddard, T. D. et al. 2018. Protein Sci, 27, 14-25)

For manuscripts utilizing custom algorithms or software that are central to the research but not yet described in published literature, software must be made available to editors and reviewers. We strongly encourage code deposition in a community repository (e.g. GitHub). See the Nature Research guidelines for submitting code & software for further information.

Data

Policy information about availability of data

All manuscripts must include a data availability statement. This statement should provide the following information, where applicable:

- Accession codes, unique identifiers, or web links for publicly available datasets
- A list of figures that have associated raw data
- A description of any restrictions on data availability

All subtomogram averaging maps were deposited in the Electron Microscopy Data Bank (EMDB) with accession code: EMD-11959 (ribosome), EMD-11958 (ATP synthase), EMD-11961 (LPOR in membrane tubes from class 1), EMD-11960 (LPOR in membrane tubes from class 2), EMD-11963 (LPOR class 1 subboxes), and EMD-11962 (LPOR class 2 subboxes).

Field-specific reporting

Please select the one below that is the best fit for your research. If you are not sure, read the appropriate sections before making your selection.

☒ Life sciences ☐ Behavioural & social sciences ☐ Ecological, evolutionary & environmental sciences

For a reference copy of the document with all sections, see nature.com/documents/nr-reporting-summary-flat.pdf

Life sciences study design

All studies must disclose on these points even when the disclosure is negative.

Sample size	Etioplasts from pea and maize were analysed from 28 and 11 independent preparations respectively. 7 tomograms were used for subtomogram averaging of ribosomes and ATP synthase, 17 for subtomogram averaging of LPOR. The number of particles was sufficient for averaging, and confirmed the reproducibility of the results.
Data exclusions	65 tomograms were reconstructed, but only 24 were used for subtomogram averaging. The selection was based on: i) quality of tilt series alignment, assessed with a standard procedure through IMOD's alignment score; ii) specimen thickness, assessed by visual inspection of the tomogram.
Replication	The micrographs, tomographic reconstructions, and experimental results in all figures are representative of at least three independent replicates. The tomograms used for subtomogram averaging analysis were collected on grids prepared from 3 (pea) and 5 (corn) independent purifications. Each one of these tomograms was collected on a distinct etioplast, and thus is a biological replicate. All attempts to reproduce the results were successful.
Randomization	Etioplast inner membranes on the cryo-EM grid were selected randomly for data acquisition.
Blinding	This work is not a case-control study, blinding is not relevant.

Reporting for specific materials, systems and methods

We require information from authors about some types of materials, experimental systems and methods used in many studies. Here, indicate whether each material, system or method listed is relevant to your study. If you are not sure if a list item applies to your research, read the appropriate section before selecting a response.

Materials & experimental systems

n/a	Involved in the study
☐	☒ Antibodies
☒	☐ Eukaryotic cell lines
☒	☐ Palaeontology and archaeology
☒	☐ Animals and other organisms
☒	☐ Human research participants
☒	☐ Clinical data
☒	☐ Dual use research of concern

Methods

n/a	Involved in the study
☒	☐ ChIP-seq
☒	☐ Flow cytometry
☒	☐ MRI-based neuroimaging

Antibodies

Antibodies used	Agrisera (Vännäs, Sweden): Anti-POR (AS05 067) and Anti-ATP synthase (AS08 370)<br>SouthernBiotech (Birmingham (AL), U.S.A.): Goat anti-rabbit secondary antibody (4055-05)<br>Jackson ImmunoResearch Europe Ltd (Ely, UK): gold-conjugated AffiniPure goat anti-rabbit secondary antibody (111-205-144)

Validation

(source: www.agrisera.com)
Anti-POR (AS05 067), tested for western blot, confirmed reactivity in A. thaliana, Cyanobacteria, H. vulgare, N. tabacum, O. sativa, P. Sogo Yukidian cultivar V3, P. sativum, T. aestivum. No exceptions from predicted reactivity were confirmed.
Related publications: Dogra et al. (2019), doi: 10.1038/s41467-019-10760-6; Zhang et al. (2018), doi: 10.1016/j.niox.2018.03.001; Han et al. (2015), doi: 10.1111/tpj.12889.

(source: www.agrisera.com)
Anti-ATP synthase (AS08 370), tested for western blot, confirmed reactivity in A. thaliana, C. reinhardtii, S. oleracea, P. sativum, E. ophiuriudes, P. vaginatum. No exceptions from predicted reactivity were confirmed. Additional notes: The prominent reaction is to a doublet around 55-58 kDa which is alpha and beta and weaker reaction to gamma subunit which is around 38 kDa.
Related publications: Du et al. (2018), doi: 10.1105/tpc.17.00446; Liu et al. (2011), doi: 10.1016/j.jplph.2011.08.019.

(source: www.southernbiotech.com):
Goat anti-rabbit secondary antibody (4055-05), tested for western blot, confirmed reactivity against rabbit IgG.
Related publications: Wu and Ma (2020), doi: 10.1016/j.archoralbio.2020.104930

(source: www.jacksonimmuno.com, www.citeab.com)
gold-conjugated AffiniPure goat anti-rabbit secondary antibody (111-205-144), tested for immuno-electron microscopy, confirmed reactivity against rabbit IgG.
Related publications: Hamada et al. (2018), doi:10.1242/jcs.216051; Mariotti et al. (2018), doi: 10.1038/s41467-017-02642-6; Farley et al. (2015), doi: 10.1016/j.neuroscience.2015.07.062
