# Supplementary figures and images for: Molecular landscape of etioplast inner membranes in higher plants
Source: Nat Plants. 2021 Apr 19;7(4):514–23. doi: 10.1038/s41477-021-00896-z (PMC8055535; doi:10.1038/s41477-021-00896-z)

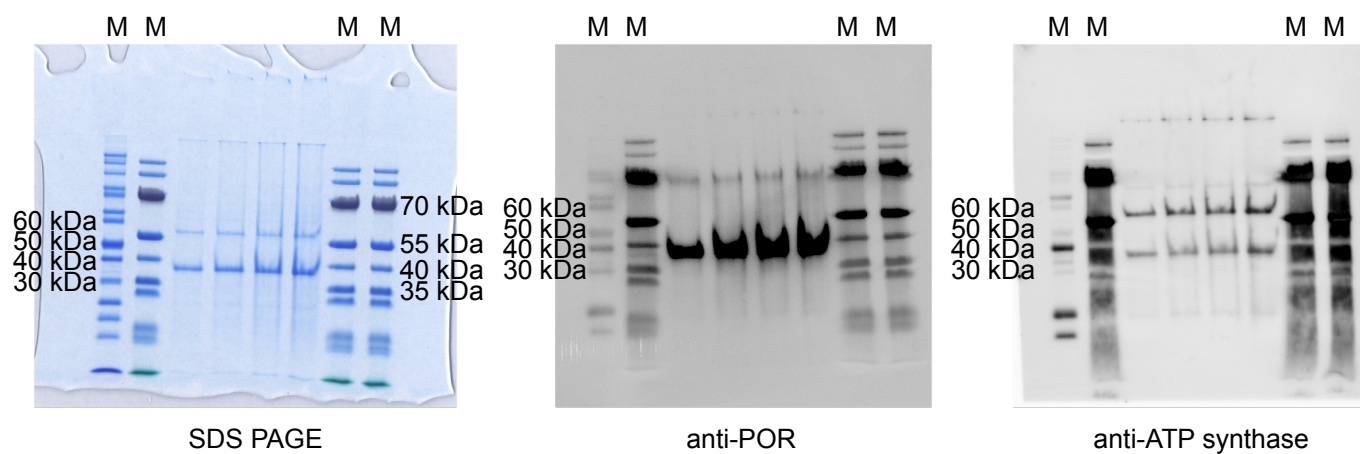

**Source Data Figure 1 | Full scans of the SDS-PAGE gel and Western blots from Extended Data Fig. 6b.**

Supplement: Source Data Fig. 1 — Unprocessed immunoblots and gels. [file 41477_2021_896_MOESM3_ESM.pdf]
